# Reducing antibiotic prescriptions for respiratory tract infections in family practice: results of a cluster randomized controlled trial evaluating a multifaceted peer-group-based intervention

**DOI:** 10.1038/npjpcrm.2015.83

**Published:** 2016-02-04

**Authors:** Marcia Vervloet, Marianne A Meulepas, Jochen W L Cals, Mariëtta Eimers, Lucas S van der Hoek, Liset van Dijk

**Affiliations:** 1 NIVEL, Netherlands Institute for Health Services Research, Utrecht, The Netherlands; 2 Dutch Institute for Rational Use of Medicine, Utrecht, The Netherlands; 3 Meetpunt Kwaliteit, Eindhoven, The Netherlands; 4 Department of Family Medicine, CAPHRI School for Public Health and Primary Care, Maastricht University, Maastricht, The Netherlands

## Abstract

Irrational antibiotic use for respiratory tract infections (RTI) is a major driver of bacterial resistance. The aim of this study was to evaluate the effect of a multifaceted peer-group based intervention aiming to reduce RTI-related antibiotic prescriptions in family practice. This was a cluster randomized controlled trial with pre- and follow-up measurement. The intervention was implemented through PharmacoTherapy Audit Meetings (PTAM) in which family physicians (FPs) and pharmacists collaborate. Four PTAM groups received the intervention consisting of: (1) FP communication skills training, including communication about delayed prescribing; (2) implementation of antibiotic prescribing agreements in FPs’ Electronic Prescribing Systems; (3) quarterly feedback figures for FPs. Four other PTAM groups were matched controls. Primary outcome measure was the number of RTI-related antibiotic prescriptions after the intervention, assessed with multilevel linear regression analyses. Total number and number of prescriptions stratified by age (under/over 12 years) were analysed. At baseline, the average total number of RTI-related antibiotic prescriptions per 1,000 patients was 207.9 and 176.7 in the intervention and control PTAM groups, respectively. At follow-up, FPs in both the intervention and control groups prescribed significantly less antibiotics. For adolescents and adults, the drop in number of antibiotic prescription was significantly larger in the intervention groups (−27.8 per 1,000 patients) than the control groups (−7.2 per 1,000 patients; *P*<0.05). This multifaceted peer-group-based intervention was effective in reducing the number of RTI-related antibiotic prescriptions for adolescents and adults. To affect antibiotic prescribing in children other methods are needed.

## Introduction

Antibiotic resistance is a major public health problem.^1–3^ A major risk factor is irrational antibiotic use, e.g., in patients with common respiratory tract infections (RTI). Limited evidence is found for the benefit of antibiotics in RTI.^4–6^ However, patients presenting with RTI symptoms are still often prescribed antibiotics in family practice.^7^ Although family physicians (FPs) are aware that they tend to overprescribe,^8,9^ non-clinical factors such as time pressure, perceived patients’ expectations and the desire to maintain good relationships are mentioned as drivers to prescribe antibiotics.^9,10^ However, patients seem to value information and/or reassurance as more important than an antibiotic prescription.^8,11,12^ Thus, the quality of communication between FPs and patients is a critical factor for optimal care. FPs need skills to identify patients’ needs and to discuss elements of the treatment such as its natural course, the benefits and harms of antibiotic treatment and when to consult the FP.^13^

Several promising interventions that could assist FPs in reducing unwarranted antibiotic prescribing can be identified. First of all, a Dutch study showed that training FPs in communication skills can result in significantly less prescriptions for antibiotics.^13^ This was confirmed by a large European study showing similar effects of enhanced communication skills training for FPs across six European countries.^14^ Second, another effective tool for reducing antibiotic prescriptions may be the delayed prescribing strategy. When giving a delayed prescription, the FP advises the patient to collect the prescription only if symptoms persist or get worse. Previous research showed that patients who received a delayed antibiotic prescription were less likely to collect their prescription at the pharmacy.^15–17^ In some trials patients in the delayed prescribing arm had more symptoms, which imply that patients are willing to tolerate some symptoms to avoid antibiotics.^16,17^ Third, in addition to improving communication skills and using the delayed prescription strategy, providing FPs with decision support can be useful both in the consultation room and as a reflection on their own prescribing behaviour.^18^ During the consultation, the Electronic Prescription System (EPS) can be helpful as it is a means to compose prescriptions based on indications, treatment regimens and treatment choices. Providing FPs with feedback figures has had some additional effects on their prescribing behaviour.^19,20^ Interventions consisting of multiple components (multifaceted), one of which focusses on communication skills, are the most effective in reducing antibiotic prescribing.^21,22^

Most interventions tackle irrational antibiotic prescribing at the individual prescriber level. The Netherlands, however, has an extensive network of PharmacoTherapy Audit Meeting (PTAM) groups. PTAMs are a series of regular meetings between FPs and pharmacists in the same catchment area during which information and views about pharmacotherapy are exchanged with the aim of improving the prescribing and dispensing of drugs.^23,24^ An increasing number of these groups operate at a high level by making formulary agreements and evaluating these agreements. This existing peer-group structure is likely to be a facilitator for the implementation of promising multifaceted interventions targeting inappropriate prescribing.

The aim of this study was thus to evaluate the effect of a PTAM-based multifaceted intervention, which consisted of (1) FP communication skills training, including communication about delayed prescribing, (2) implementation of PTAM group’s antibiotic prescribing agreements in the FPs’ EPS and (3) providing quarterly feedback figures of RTI-related antibiotic prescriptions to the participating FPs, on the volume of RTI-related antibiotic prescriptions in family practice.

## Results

### Sample characteristics

Four intervention PTAM groups with 39 FPs and 4 matched control PTAM groups with 38 FPs participated in the study. Table 1 presents the baseline characteristics of the PTAM groups and the number of RTI-related antibiotic prescriptions per 1,000 patients at baseline and follow-up. The number of prescriptions decreased in all intervention PTAM groups after the intervention. Two control PTAM groups also showed a large decrease in prescriptions, whereas the other two control PTAM groups showed no or a minimal decrease. Doxycycline and amoxicillin were the most frequently prescribed antibiotics during baseline and follow-up.

### Intervention effects on the total number of antibiotic prescriptions

At baseline, the average number of RTI-related antibiotic prescriptions issued in the intervention and control PTAM groups did not significantly differ (Table 2). At follow-up, a significant decrease in prescriptions was observed in both the intervention and control groups. The reduction in the intervention groups was not larger than that in the control groups (*P*=0.09). Most variation could be attributed to the level of the FP (81%), indicating that differences in antibiotic prescribing between FPs were much larger than differences between PTAM groups.

### Intervention effects stratified by age

For patients younger than 12 years, significantly less RTI-related antibiotic prescriptions were issued at follow-up in both the intervention and control PTAM groups (Table 2). The reduction in the intervention groups was not larger than that in the control groups (*P*=0.08). For patients of 12 years and older, however, the drop in number of antibiotic prescription was significantly larger in the intervention groups (−27.8 per 1,000 patients) than the control groups (−7.2 per 1,000 patients; *P*<0.05). About 79–82% of the variation in antibiotic prescribing could be attributed to the level of the FP, 6–10% to the PTAM level.

### Delayed prescriptions

FPs in the intervention PTAM groups issued 160 delayed prescriptions during a period of 4 and a half months (one PTAM group) up to 6 months (three PTAM groups; Table 3). These delayed prescriptions were mostly prescribed for common cold/rhinosinusitis (43%), cough (34%), otitis media (17%) and sore throat (6%). The number of delayed prescriptions collected at the pharmacy varied largely between the PTAM groups. For children younger than 12, the range was 9–61% (mean 43%). For adolescents and adults, this range was 11–55% (mean 34%).

## Discussion

### Main findings

The aim of this study was to evaluate the effect of a multifaceted peer-group-based intervention, including communication skills training for FPs, implementation of formulary agreements in FPs’ EPS, and providing FPs with feedback on prescription figures on the volume of RTI-related antibiotics prescriptions in the Dutch primary care setting. This study shows that the intervention resulted in a significantly larger drop in number of antibiotic prescriptions issued for patients aged 12 years and older. The intervention had no effect on antibiotic prescribing for children younger than 12 years.

### Interpretation of findings in relation to previously published work

Although the drop in antibiotic prescriptions for adolescents and adults was significantly larger in the intervention PTAM groups, the control groups issued significantly less antibiotic prescriptions at follow-up as well. One explanation might be that the two control PTAMs where this reduction was observed might already have been more susceptible to the antibiotic resistance problem. At national and international level, more and more attention is being paid to the resistance problem.^25,26^ This may have already triggered FPs to reconsider their antibiotic prescribing behaviour. An increased awareness because of their participation in this study (Hawthorne effect^27^), as they were included as a ‘waiting list control group’, might be another explanation.

A previous Dutch study in which a comparable intervention was evaluated (communication skills training for FPs and feedback on prescribing behaviour, but not formulary agreements implemented in the EPS or delayed prescribing) also found reduced prescribing rates.^28^ Whereas this previous study only studied the change in total number of antibiotic prescriptions, our study showed that it is important to take the patient’s age into account as prescription rates only dropped in adolescents and adults. In addition, previous research has shown that FPs who are provided with decision support during the consultation, like formulary agreements in our intervention, more often prescribe according to the given advice,^29^ suggesting that making formulary agreements and implementing them in the EPS is a strong component of our intervention. Furthermore, the two diagnoses for which the FPs in our study most frequently issued a delayed prescription (common cold/rhinosinusitis and cough) amount to a considerable share of all antibiotic prescriptions issued in family practice. This suggests that providing delayed prescriptions for RTI can be used to reduce the volume of antibiotics prescribed, which is in line with the findings of a recent review on delayed prescribing for RTI.^30^

Our intervention was not successful in reducing RTI-related antibiotic prescriptions in children under the age of 12. An explanation for this might be that our intervention was aimed at the FP and the collaboration with the pharmacist. A recent review found that interventions targeting both parents and prescribers were most successful in reducing antibiotic prescribing in children with RTI.^31^ In addition, besides providing parents with a delayed prescription, engaging not only parents but also children and providing information focused on specific symptoms rather than generic information seem to be successful strategies for this purpose.^32^ Furthermore, our intervention mainly focused on acute cough and rhinosinusitis. The latter is very uncommon in young children, and this group actually consults most often for RTI-related symptoms. As the communication skills training was mostly focused on traditional one-on-one consultations, consultations for childhood infections may require specific consultation skills due to parental expectations and public beliefs on antibiotics for childhood fever.^33,34^

Our study showed a large variation between individual FPs in antibiotic prescribing behaviour rather than between PTAM groups, confirming results from earlier research on the existence of practice variation in drug prescribing.^7,35^ These findings implicate that the PTAM structure might not be the most successful way to implement this intervention aimed at changing FPs’ individual prescribing behaviour. On the other hand, the structure allows for an easy implementation of the intervention as FPs and pharmacists are already brought together in these meetings and are committed to optimising pharmacotherapy, which is the goal of these PTAMs.^23^ With more than 800 PTAM groups in the Netherlands, this network is extensive. Furthermore, peer-review (and peer pressure) and making prescribing agreements during the PTAMs might support behaviour change.

### Strengths and limitations of the study

The effect of the intervention might be underestimated because of power issues. The small number of PTAM groups (*n*=8) might cause significant differences between the intervention and control PTAM groups to remain undetected. Using a more simplistic analysis design instead of multilevel analyses yielded the same results. However, the clinical relevance of the decrease in the volume of antibiotic prescriptions, whether statistically significant or not, is of much more importance.

Despite the matching procedure used in assigning PTAMs to the intervention or control group, the total FP practice size varied largely between PTAM groups. Overall, the intervention PTAM groups had smaller practice sizes than their controls. The variability in practice size conceals the intervention effect as it complicates estimation of the variability that can be attributed to the effect of the intervention. By standardizing the number of prescriptions (per 1,000 patients), this variability was reduced, providing a more precise estimation of the intervention effect. As the practice size of each individual FP was not available, standardisation was done at PTAM level. This can be justified because of our focus on the reduction in antibiotic prescriptions at PTAM level.

No information was available on the age, gender and years in practice of the participating FPs. However, within each PTAM group, the distribution in these FP characteristics is a representative reflection of that of the FPs working in that region at that moment. As the whole PTAM group participated, there appears to be no selection of participating FPs within groups.

The number of antibiotics prescribed for RTI is based on the number of antibiotics dispensed at the pharmacy. This might have introduced an inaccuracy as these numbers do not always exactly correspond, e.g., when patients receive an antibiotic prescription from their FP, but never collect the prescription at the pharmacy.

Our intervention consisted of three components offered simultaneously. Therefore, it remains unclear whether it is the combination of components or a single component that changed FPs’ prescribing behaviour. Although multifaceted interventions appear to be most promising in reducing antibiotic prescriptions,^21,22^ it would be interesting to explore the effect of the individual components. Qualitative research can provide insight into FPs’ behaviour change in antibiotic prescribing due to complex interventions.^36^

### Implications for future research, policy and practice

Due to power issues and large variability in practice sizes, the effect of the intervention might be underestimated. Therefore, it is recommended to repeat this study with a larger number of PTAM groups (and consequently a larger number of participating FPs) and to also match intervention and control PTAM groups based on practice size. As our intervention did not affect antibiotic prescribing in children younger than 12, further research on incorporating engagement of parent and child in the intervention is suggested as this might be a strategy to reduce antibiotic prescriptions for RTI in children. Delayed prescribing was very infrequently used by FPs, which merits further exploration of the attitudes of FPs on this prescribing strategy and barriers to use it.

### Conclusions

Our multifaceted peer-group-based intervention including communication skills training for FPs, implementation of formulary agreements in the FPs’ EPS, and regular discussion of prescription feedback figures, showed promising results for reducing the number of RTI-related antibiotic prescriptions for adolescents and adults, but not for children younger than 12. Other methods need to be explored to reduce antibiotic prescribing in children.

## Materials and methods

### Design

This was a cluster randomized controlled trial with a baseline and follow-up measurement. The study was conducted in North-Limburg, a region in the southeast of the Netherlands. Eight PTAM groups from this region participated. First, four couples of comparable PTAM groups were formed based upon the number of FPs in the group and whether the group was located in a rural or urban area. Next, one PTAM group of each couple was randomly assigned to the intervention group and the other to the control group. By matching the PTAM groups on size and urbanisation before randomisation, it was prevented that only the larger or only the smaller PTAM groups would be allocated to the intervention group, which would have biased the intervention effects.

### Ethical approval

This study was registered in the Netherlands National Trial Registry (trial registration number NTR2753). The study protocol was evaluated by the Medical Ethical Committee Radboud Medical Centre in Nijmegen, who decided that ethical approval was not required for this study.

### Intervention

The intervention consisted of multiple components. First, all participating FPs used the EPS. When the FP diagnosed an RTI, the EPS first suggested ‘no prescription’ and provided the FP with advice to give the patient. The next suggestion by the EPS, if the FP still wished to prescribe an antibiotic, was ‘delayed prescription’ together with information about for which patients in which situations such a prescription would be feasible. Only after these two suggestions, the FP could prescribe a ‘normal prescription’, if (s)he wished to. All FPs received technical support on how to work with the EPS to be able to adhere to antibiotic prescribing agreements made in a later stage. Hereafter, a PTAM was prepared on the two most common clinical presentations of RTI: acute cough and rhinosinusitis. During this PTAM, FPs were educated on treatment guidelines for both complaints and were trained in their communication skills. Agreements were made about antibiotic prescribing for RTI, which were implemented in their EPS. In the following PTAM, 3 months later, feedback figures on their antibiotic prescribing behaviour were discussed. The control groups did not receive any training, support or feedback on antibiotic prescribing during this period, they focused on a topic other than antibiotics. The control groups received the intervention once the study had finished.

### Participants

**Figure 1:**
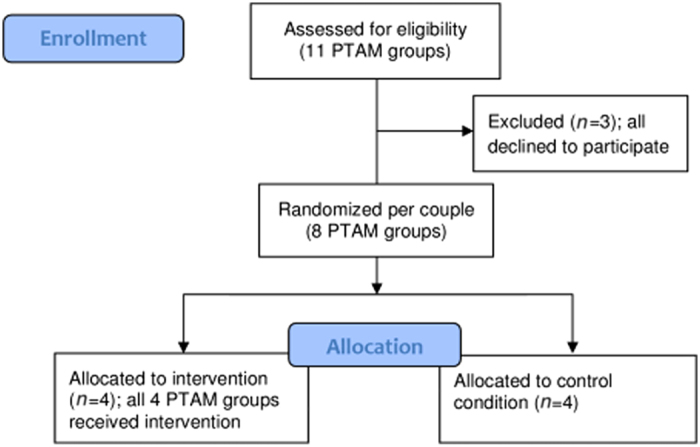
Flow diagram of PTAM groups in this study.

In the Netherlands, practically all FPs participate in PTAM groups. PTAM groups consist of a mix of FPs who either practice in a group, a duo or single practice, and the pharmacies in the same catchment region. FPs and pharmacists in the PTAM groups involved in this study were all members of Cohesie, a cooperation of 113 FPs in North-Limburg, and SANL (in Dutch: Samenwerkende Apotheken Noord-Limburg), a cooperation of pharmacies in this region. Both parties intend to improve prescribing by using the EPS as a control system for the formulary agreements made in their PTAM groups. All 11 PTAM groups from Cohesie were approached for participation (Figure 1). Three PTAM groups refused participation; two groups were already participating in other antibiotic stewardship programs and the third was not willing to start a new project because of the workload.

### Outcome measures

The primary outcome measure was the difference in the number of RTI-related antibiotic prescriptions per 1,000 patients at follow-up between the intervention and control PTAM groups. Dispensing data of RTI-related antibiotic courses were obtained from the pharmacies linked to the participating FPs. For each prescription, the prescribing FP was known, thus actual dispensed RTI-related prescriptions could be allocated to FPs in either the intervention or control PTAM groups. Antibiotic prescriptions were only provided after a consult with the FP. Prescriptions issued by physicians outside the study were not included. Baseline measurement covered the year before the intervention, including the day of the intervention. Follow-up covered the year after the training day. For two PTAM groups, an intervention group and its matched control, the follow-up covered 353 days, as no data were available for the last 12 days. In addition, the number of delayed prescriptions in the intervention groups was studied and diagnoses for delayed prescriptions were verified with the FPs.

### Sample size

Based upon figures from the Dutch National Survey in General Practice (DNSGP), we calculated that there will be about 23,500 RTI contacts in 1 year in both our intervention and control groups. Also calculated from the DNSGP, 27% results in an antibiotic prescription (*n*=6,350 consultations). A 25% decrease in the number of dispensed antibiotic prescriptions seems feasible given the results of international studies. Power calculation (*α*=0.05, power=0.90) shows that at least 800 patients are needed in each group to show such difference. With 23,500 RTI consultations in 1 year in both groups, the number of patients will be sufficient.

### Statistical analyses

Multilevel analyses were performed to analyse the effect of the intervention on the volume of RTI-related antibiotic prescriptions. The two measurements (baseline and follow-up) are clustered in FPs who in their turn are clustered in PTAM groups. Therefore, a multilevel linear regression model with three levels was used: (1) measurement, (2) FP and (3) PTAM. The standardized number of antibiotic prescriptions (per 1,000 patients per PTAM) was calculated and used in the multilevel analyses model as dependent variable. The antibiotic prescription data at FP level were standardised at PTAM level because the practice size per individual FP was not available. Whether a PTAM was assigned as an intervention or control group, was added as a factor variable (‘group’) in the model. The interaction term group×measurement was added to the model, since it was expected that the effect would differ between groups and between measurements. The model was fitted with maximum likelihood estimation. Differences in the volume of prescriptions between the control and intervention PTAM groups were tested two-tailed with the significance level set at *P*<0.05. Separate predefined multilevel analyses using the same model were performed for prescriptions for patients younger than 12 and for patients aged 12 years and older, as childhood infections might be differently treated from infections in adolescents and adults. All analyses were performed with Stata SE 12.1 (StataCorp, College Station, TX, USA).

## Figures and Tables

**Table 1:** The standardized number of antibiotic prescriptions for RTI (per 1,000 patients at PTAM group level per year) at baseline and follow-up in the intervention and matched control PTAM groups, and the change in number of prescriptions between baseline and follow-up

	*Baseline*	*Follow-up*	*Change*
	*RTI-related antibiotic prescriptions/1,000 patients/year*	*RTI-related antibiotic prescriptions/1,000 patients/year*	
*Intervention PTAM groups*			
Group 1: 5 FPs; 5,359 patients	185	118	−67 (36%)
Group 2: 5 FPs; 8,826 patients	177	144	−33 (19%)
Group 3: 13 FPs; 19,822 patients	280^a^	243	−37 (13%)
Group 4: 16 FPs; 25,476 patients	170^a^	117	−53 (31%)
			
*Matched control PTAM groups*			
Group 1: 6 FPs; 11,464 patients	173	138	−35 (20%)
Group 2: 5 FPs; 12,702 patients	205	199	−6 (3%)
Group 3: 11 FPs; 29,020 patients	190	190	0 (0%)
Group 4: 16 FPs; 41,581 patients	152	113	−39 (26%)

Abbreviations: FP, family physician; PTAM, Pharmaco Therapy Audit Meetings; RTI, respiratory tract infections.

a One FP had no antibiotic prescriptions for RTI at baseline.

**Table 2:** Differences in standardized average number of RTI-related antibiotic prescriptions (per 1,000 patients per year) between intervention and control PTAM groups for the total number of prescriptions and for prescriptions stratified by age (cut-off: 12 years), assessed with multilevel modelling

	*Intervention groups* n*/1,000 patients/year*	*Control groups* n*/1,000 patiens/year*	*Difference*
*Total number of RTI-related antibiotic prescriptions*			
Baseline	207.9	176.7	31.1
Follow-up	161.2	154.1	7.1
Change	−46.7^***^	−22.7^*^	

	*Intervention groups*^a^ n*/1,000 patients/year*	*Control groups*^b^ n*/1,000 patients/year*	*Difference*
*Number of RTI-related antibiotic prescriptions <12 years*			
Baseline	30.6	24.2	6.4
Follow-up	21.6	19.2	2.7
Change	−8.9^***^	−5.0^**^	
			
*Number of RTI-related antibiotic prescriptions ⩾12 years*			
Baseline	156.3	129.7	26.6
Follow-up	128.4	122.5	6.0
Change	−27.8^***^	−7.2	

Significance levels: *P<0.05; **P<0.01; ***P<0.001.

Abbreviations: PTAM, PharmacoTherapy Audit Meetings; RTI, respiratory tract infections.

a For 5.3% of patients age was unknown.

b For 10.6% of patients age was unknown.

**Table 3:** Number of delayed prescriptions issued by FPs in the intervention PTAM groups and those collected at the pharmacy during a maximum period of 6 months, stratified by age

*Intervention PTAM groups*	*Delayed prescription <12 years*	*Collected at pharmacy*	*Delayed prescription ⩾12 years*	*Collected at pharmacy*
	n	n *(%)*	n	n *(%)*
Group 1: 5 FPs; 5,359 patients	11	1 (9%)	9	1 (11%)
Group 2: 5 FPs; 8,826 patients	20	9 (45%)	11	6 (55%)
Group 3: 13 FPs; 19,822 patients	27	10 (37%)	15	2 (13%)
Group 4: 16 FPs; 25,476 patients	28	17 (61%)	39	16 (41%)
Total (39 FPs; 59,483 patients)	86	37 (43%)	74	25 (34%)

Abbreviations: FP, family physician; PTAM, PharmacoTherapy Audit Meetings.

## References

[bib1] Levy, S. B. Antibiotic resistance-the problem intensifies. Adv. Drug Deliv. Rev. 57, 1446–1450 (2005). 15949867 10.1016/j.addr.2005.04.001

[bib2] Livermore, D. M. Bacterial resistance: origins, epidemiology, and impact. Clin. Infect. Dis. 36, S11–S23 (2003). 12516026 10.1086/344654

[bib3] ECDC/EMEA. The Bacterial Challenge: Time to React. A Call to Narrow the Gap Between Multidrug-Resistant Bacteria in the EU and the Development of New Antibacterial. Agents ECDC/EMEA joint technical report (Stockholm, 2009).

[bib4] Smucny, J. , Fahey, T. , Becker, L. , Glazier, R. & McIsaac, W. Antibiotics for acute bronchitis. Cochrane Database Syst. Rev. 2000, CD000245 (2000). 10.1002/14651858.CD000245 11034678

[bib5] Kenealy, T. & Arroll, B. Antibiotics for the common cold and acute purulent rhinitis. Cochrane Database Syst. Rev. 6, Cd000247 (2013). 23733381 10.1002/14651858.CD000247.pub3 PMC7044720

[bib6] Zoorob, R. , Sidani, M. A. , Fremont, R. D. & Kihlberg, C. Antibiotic use in acute upper respiratory tract infections. Am. Fam. Physician 86, 817–822 (2012). 23113461

[bib7] van Dijk, L. , de Jong, J. D. , Westert, G. P. & de Bakker, D. H. Variation in formulary adherence in general practice over time (2003-2007). Fam. Pract. 28, 624–631 (2011). 21788374 10.1093/fampra/cmr043

[bib8] Butler, C. C. , Rollnick, S. , Pill, R. , Maggs-Rapport, F. & Stott, N. Understanding the culture of prescribing: qualitative study of general practitioners' and patients' perceptions of antibiotics for sore throats. Br. Med. J. 317, 637–642 (1998). 9727992 10.1136/bmj.317.7159.637 PMC28658

[bib9] Ackerman, S. L. , Gonzales, R. , Stahl, M. S. & Metlay, J. P. One size does not fit all: evaluating an intervention to reduce antibiotic prescribing for acute bronchitis. BMC Health Serv. Res. 13, 462 (2013). 24188573 10.1186/1472-6963-13-462 PMC4228248

[bib10] Little, P. et al. Importance of patient pressure and perceived pressure and perceived medical need for investigations, referral, and prescribing in primary care: nested observational study. Br. Med. J. 328, 444 (2004). 14966079 10.1136/bmj.38013.644086.7C PMC344266

[bib11] Welschen, I. , Kuyvenhoven, M. , Hoes, A. & Verheij, T. Antibiotics for acute respiratory tract symptoms: patients' expectations, GPs' management and patient satisfaction. Fam. Pract. 21, 234–237 (2004). 15128681 10.1093/fampra/cmh303

[bib12] Shapiro, E. Injudicious antibiotic use: an unforeseen consequence of the emphasis on patient satisfaction? Clin. Ther. 24, 197–204 (2002). 11833832 10.1016/s0149-2918(02)85015-9

[bib13] Cals, J. W. , Butler, C. C. , Hopstaken, R. M. , Hood, K. & Dinant, G. J. Effect of point of care testing for C reactive protein and training in communication skills on antibiotic use in lower respiratory tract infections: cluster randomised trial. Br. Med. J. 338, b1374 (2009). 19416992 10.1136/bmj.b1374 PMC2677640

[bib14] Little, P. et al. Effects of internet-based training on antibiotic prescribing rates for acute respiratory-tract infections: a multinational, cluster, randomised, factorial, controlled trial. Lancet 382, 1175–1182 (2013). 23915885 10.1016/S0140-6736(13)60994-0 PMC3807804

[bib15] Little, P. et al. Open randomised trial of prescribing strategies in managing sore throat. Br. Med. J. 314, 722–727 (1997). 9116551 10.1136/bmj.314.7082.722 PMC2126131

[bib16] Arroll, B. , Kenealy, T. & Kerse, N. Do delayed prescriptions reduce antibiotic use in respiratory tract infections? A systematic review. Br. J. Gen.Pract. 53, 871–877 (2003). 14702908 PMC1314731

[bib17] Spurling, G. K. , Del Mar, C. B. , Dooley, L. & Foxlee, R. Delayed antibiotics for symptoms and complications of respiratory infections. Cochrane Database Syst. Rev. 2004, CD004417 (2004). 10.1002/14651858.CD004417.pub2 15495108

[bib18] McCullough, J. M. , Zimmerman, F. J. & Rodriguez, H. P. Impact of clinical decision support on receipt of antibiotic prescriptions for acute bronchitis and upper respiratory tract infection. J. Am. Med. Inform. Assoc. 21, 1091–1097 (2014). 25002458 10.1136/amiajnl-2014-002648 PMC4215050

[bib19] Dormuth, C. R. , Carney, G. , Taylor, S. , Bassett, K. & Maclure, M. A randomized trial assessing the impact of a personal printed feedback portrait on statin prescribing in primary care. J. Contin. Educ. Health Prof. 32, 153–162 (2012). 23008077 10.1002/chp.21140

[bib20] Forsetlund, L. et al. Continuing education meetings and workshops: effects on professional practice and health care outcomes. Cochrane Database Syst. Rev. 2009, CD003030 (2009). 10.1002/14651858.CD003030.pub2 PMC7138253 19370580

[bib21] Arnold, S. R. & Straus, S. E. Interventions to improve antibiotic prescribing practices in ambulatory care. Cochrane Database Syst. Rev. Cd003539, (4) (2005). 16235325 10.1002/14651858.CD003539.pub2 PMC7003679

[bib22] van der Velden, A. W. et al. Effectiveness of physician-targeted interventions to improve antibiotic use for respiratory tract infections. Br. J. Gen. Pract. 62, e801–e807 (2012). 23211259 10.3399/bjgp12X659268 PMC3505412

[bib23] Florentinus, S. R. et al. Which pharmacists contribute to high-level pharmacotherapy audit meetings with general practitioners? Ann. Pharmacother. 40, 1640–1646 (2006). 16912245 10.1345/aph.1H180

[bib24] van Dijk, L. & de Bakker, D. H. Professionalization of Dutch PRGs and volume and costs of frequently prescribed drugs. J. Public Health 10, 292–304 (2002).

[bib25] Ministery of Health WaS. Parliamentary document 'Antibiotic resistance' 2 July 2013 [In Dutch: Antibioticaresistentie. Kamerbrief 2 juli 2013, kenmerk 124315-105291-PG] (2013).

[bib26] TATFAR. Transatlantic Taskforce on Antimicrobial Resistance (TATFAR): progress report. Recommendations for future collaboration between the US and EU. Available at http://www.cdc.gov/drugresistance/pdf/TATFAR-Progress_report_2014.pdf (2014).

[bib27] Braunholtz, D. A. , Edwards, S. J. & Lilford, R. J. Are randomized clinical trials good for us (in the short term)? Evidence for a ‘trial effect’. J. Clin. Epidemiol. 54, 217–224 (2001). 11223318 10.1016/s0895-4356(00)00305-x

[bib28] Welschen, I. , Kuyvenhoven, M. M. , Hoes, A. W. & Verheij, T. J. Effectiveness of a multiple intervention to reduce antibiotic prescribing for respiratory tract symptoms in primary care: randomised controlled trial. Br. Med. J. 329, 431 (2004). 15297305 10.1136/bmj.38182.591238.EB PMC514206

[bib29] de Jong, J. D. , Groenewegen, P. P. , Spreeuwenberg, P. , Westert, G. P. & de Bakker D. H. Do decision support systems influence variation in prescription? BMC Health Serv. Res. 9, 20 (2009). 19183464 10.1186/1472-6963-9-20 PMC2662826

[bib30] Spurling, G. K. , Del Mar, C. B. , Dooley, L. , Foxlee, R. & Farley, R. Delayed antibiotics for respiratory infections. Cochrane Database Syst. Rev. 4, Cd004417 (2013). 23633320 10.1002/14651858.CD004417.pub4

[bib31] Vodicka, T. A. et al. Reducing antibiotic prescribing for children with respiratory tract infections in primary care: a systematic review. Br. J. Gen. Pract. 63, e445–e454 (2013). 23834881 10.3399/bjgp13X669167 PMC3693801

[bib32] Andrews, T. et al. Interventions to influence consulting and antibiotic use for acute respiratory tract infections in children: a systematic review and meta-analysis. PLoS ONE 7, e30334 (2012). 22299036 10.1371/journal.pone.0030334 PMC3267713

[bib33] de Bont, E. G. , Francis, N. A. , Dinant, G. J. & Cals, J. W. Parents' knowledge, attitudes, and practice in childhood fever: an internet-based survey. Br. J. Gen. Pract. 64, e10–e16 (2014). 24567577 10.3399/bjgp14X676401 PMC3876146

[bib34] Walsh, A. , Edwards, H. & Fraser, J. Influences on parents' fever management: beliefs, experiences and information sources. J. Clin. Nurs. 16, 2331–2340 (2007). 17419783 10.1111/j.1365-2702.2006.01890.x

[bib35] de Bakker, D. H. , Coffie, D. S. , Heerdink, E. R. , van Dijk, L. & Groenewegen, P. P. Determinants of the range of drugs prescribed in general practice: a cross-sectional analysis. BMC Health Serv. Res. 7, 132 (2007). 17711593 10.1186/1472-6963-7-132 PMC2045668

[bib36] Cals, J. W. , Butler, C. C. & Dinant, G. J. 'Experience talks': physician prioritisation of contrasting interventions to optimise management of acute cough in general practice. Implement. Sci. 4, 57 (2009). 19737382 10.1186/1748-5908-4-57 PMC2742510

